# Calcium and/or Vitamin D Supplementation for the Prevention of Fragility Fractures: Who Needs It?

**DOI:** 10.3390/nu12041011

**Published:** 2020-04-07

**Authors:** Ian R Reid, Mark J Bolland

**Affiliations:** 1 Department of Medicine, Faculty of Medical and Health Sciences, University of Auckland, Auckland 1142, New Zealand; m.bolland@auckland.ac.nz; 2 Auckland District Health Board, Auckland 1051, New Zealand

**Keywords:** calcium, vitamin D, osteoporosis, bisphosphonate

## Abstract

Vitamin D and calcium have different biological functions, so the need for supplementation, and its safety and efficacy, need to be evaluated for each separately. Vitamin D deficiency is usually the result of low sunlight exposure (e.g., in frail older people, those who are veiled, those with dark-skin living at higher latitudes) and is reversible with calciferol 400–800 IU/day. Calcium supplements produce a 1% increase in bone density in the first year of use, without further increases subsequently. Vitamin D supplements do not improve bone density in clinical trials except in analyses of subgroups with baseline levels of 25-hydroxyvitamin D <30 nmol/L. Supplementation with calcium, vitamin D, or their combination does not prevent fractures in community-dwelling adults, but a large study in vitamin D-deficient nursing home residents did demonstrate fracture prevention. When treating osteoporosis, co-administration of calcium with anti-resorptive drugs has not been shown to impact on treatment efficacy. Correction of severe vitamin D deficiency (<25 nmol/L) is necessary before use of potent anti-resorptive drugs to avoid hypocalcemia. Calcium supplements cause gastrointestinal side effects, particularly constipation, and increase the risk of kidney stones and, probably, heart attacks by about 20%. Low-dose vitamin D is safe, but doses >4000 IU/day have been associated with more falls and fractures. Current evidence does not support use of either calcium or vitamin D supplements in healthy community-dwelling adults.

## 1. Introduction

Calcium and vitamin D are often discussed together as interventions for promoting bone health, but it is important to remember that they are quite distinct entities that play different roles in mineral metabolism, have different indications for their therapeutic use, and different safety profiles when used as supplements.

Calcium is a key raw material for the laying down of bone. Together with phosphate, it makes up the mineral component of bone, which is laid down within the collagen scaffold constructed by the osteoblasts. Calcium has other critically important physiological roles, particularly in nerve function, muscle contraction, the electrophysiology of the heart, intracellular signaling, and coagulation, so maintenance of a stable extracellular calcium concentration is a high homeostatic priority. Increasing calcium intake would only be expected to benefit bone health if calcium supply was a limiting factor impacting on either the density or architecture of bone.

Vitamin D is a complex organic molecule derived from cholesterol. It is formed in human skin as a result of ultraviolet light exposure. It is biologically inactive until hydroxylated at two sites. The activation of vitamin D is subject to precise homeostatic regulation since this is a key element of the regulation of circulating calcium levels. Activated vitamin D contributes to the maintenance of serum calcium levels by increasing the absorption of calcium in the upper small bowel and by stimulating osteoclastic bone resorption. Activated vitamin D also stimulates intestinal absorption of phosphate. Regulatory systems exist to prevent both hypercalcemia and hyperphosphatemia, since either could result in soft tissue calcification with consequent damage to the tissues affected.

## 2. Effects of Calcium Supplements

Ingestion of a calcium supplement, typically 500 or 1000 mg, results in an influx of calcium ions into the circulation. This results in an increase in serum calcium towards the upper end of the normal range, leading to modest suppression of parathyroid hormone and of bone resorption [1]. The effects on resorption appear to persist for only a few months, but bone formation markers and parathyroid hormone levels are suppressed by 10–20% for as long as supplements are continued [2].

Meta-analysis of the many trials that have assessed the effects of calcium supplementation on bone density demonstrates that there is a benefit of about 1% in the supplement group at the end of the first year of use, which does not increase further with continued supplementation [3]. This small, non-cumulative change in bone density appears to result from a decrease in the remodeling space (i.e., a reduction in the number of osteoclastic resorption cavities spread across the bone surface). However, there is no sustained change in the balance between bone formation and bone resorption. The bone density effects of increased calcium intake are similar for dietary or supplement sources, are independent of co-administration of vitamin D, and are unrelated to baseline dietary calcium intake and the dose of calcium used [3].

### 2.1. Fractures

Most observational studies do not show a relationship between calcium intake and fracture risk [4]. A global view of fracture epidemiology is consistent with this, with low calcium intake regions (such as Africa and Asia) historically having lower fracture rates than regions where dairy products form an important part of the diet (e.g., Western Europe and North America). This has sometimes been referred to as the “calcium paradox”, though it is only paradoxical if one assumes that intake and fracture risk are inversely related.

Zhao has recently meta-analyzed the 33 trials of calcium supplements in community-dwelling participants (*n* = 51,145) reporting fracture endpoints [5]. Monotherapy with calcium tended to increase hip fracture risk (relative risk, 1.53; 95% confidence interval, 0.97, 2.42), and when combined with vitamin D was without effect. Calcium alone or with vitamin D is without effect on all other fracture endpoints. Outcomes were unrelated to gender, calcium dose, dietary calcium intake, or fracture history.

This analysis omitted a substantial trial of calcium plus vitamin D carried out in elderly nursing home residents because the participants were not “community-dwelling” [6,7,8]. In this study of 3270 women, hip fracture and non-vertebral fractures were both significantly reduced by about 30% [6,7]. Thus, these results appear to be quite distinct from those found in community-dwelling adults. This different outcome is likely to be related to the markedly deficient 25-hydroxyvitamin D levels at baseline (≤20 nmol/L) in the nursing home women, probably sufficient to cause osteomalacia in many of the trial participants. This highlights the problem of sunlight deprivation in frail, institutionalized, elderly people, and the importance of providing vitamin D supplements to mitigate this. However, the bone density and fracture improvements seen with calcium plus vitamin D supplementation in this population are clearly not reproduced in community-dwelling adults who enter trials with much higher 25-hydroxyvitamin D levels. The principal trials assessing the effect of calcium on fracture are set out in Table 1.

**Figure 1:**
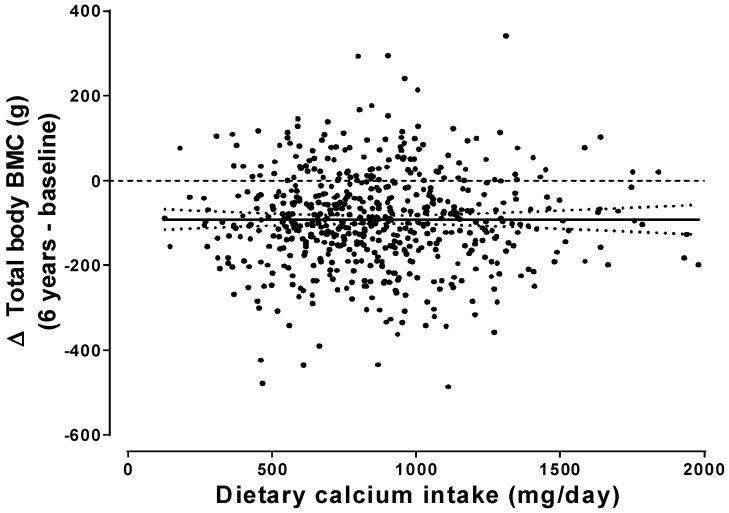
Absolute change (Δ) in total body bone mineral content (BMC) over 6 years in 698 osteopenic postmenopausal women not receiving bone-active medications, in relation to each woman’s average calcium intake assessed at baseline, year 3, and year 6. The regression line (with 95% CIs) for this relationship is shown (*p* = 0.99). From Bristow et al., *J Clin Endocrinol Metab*, 2019 [12]. Used with permission.

The failure of increased calcium intake to impact significantly on either bone density or fracture is, at first, surprising in view of the strong, positive relationship between calcium intake and calcium balance demonstrated in studies from the 1970s, which led to recommendations for calcium intakes of 1500 mg/day in postmenopausal women [9]. However, subsequent more rigorous analyses of balance studies [10] did not support those recommendations, and recent studies over periods up to 6 years show no dependence of *bone* balance on calcium intake (Figure 1) [11,12,13]. Thus, calcium balance assessed over a week or two is not an adequate surrogate for long-term changes in bone mass, and the shortcomings of calcium balance studies have resulted in an unjustified emphasis on calcium intake in osteoporosis prevention and treatment.

### 2.2. Safety

Users and prescribers of calcium supplements will be well aware of their propensity to cause gastrointestinal upset, particularly constipation. The latter can be a major issue in frail elderly people who are already prone to this problem. Gastrointestinal problems arising from the use of calcium supplements are not always trivial, and one clinical trial showed that the frequency of acute admissions to hospital for abdominal problems is doubled in those randomized to calcium [14]. Most of the calcium absorbed from a supplement is passed out again in the urine, so the 17% increase in risk of urinary calculi found in the Women’s Health Initiative is not surprising [15]. There is evidence that calcium supplements increase the risk of myocardial infarction and, possibly, stroke [16].Trials in nephrology patients, using calcium as a phosphate binder, also demonstrate increased mortality in those receiving calcium [17]. As noted above, calcium supplementation acutely elevates serum calcium concentration, and higher serum calcium levels have been associated in cohort studies with increased risk of myocardial infarction, stroke, and death [18]. This is complemented by two recent Mendelian randomization studies showing that small increases in circulating calcium concentrations within the normal range are associated with increased risks of vascular disease [19,20]. These findings provide a plausible mechanism for how calcium supplements might increase cardiovascular disease.

## 3. Effects of Vitamin D Supplements

While the immediate biochemical responses to a calcium supplement are largely independent of baseline calcium intake, responses to dosing with vitamin D depend strongly on the baseline level of 25-hydroxyvitamin D. In individuals with 25-hydroxyvitamin D levels >30 nmol/L, supplementation results only in small, dose-related increases in levels of that metabolite. However, in individuals with 25-hydroxyvitamin D <30 nmol/L, supplementation results in substantial increases in 25-hydroxyvitamin D, correction of secondary hyperparathyroidism, normalization of serum calcium and phosphate, and reduction in bone turnover [21].

**Figure 2:**
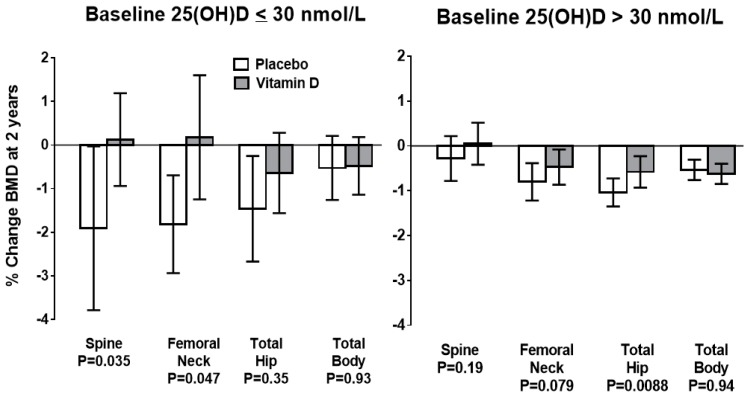
Changes in bone mineral density (BMD) from baseline to 2 years in the vitamin D and placebo groups of the ViDA Study, grouped according to baseline serum 25-hydroxyvitamin D (25(OH)D) concentrations. Data are mean ± 95% confidence intervals. P values for between-groups comparisons are shown. From: Reid IR et al., Effect of monthly high-dose vitamin D on bone density in community-dwelling older adults: substudy of a randomized controlled trial. *J Int Med* 282: 452-460, 2017 [24]. Used with permission.

Meta-analyses of the effect of vitamin D supplements on bone density show no clinically significant benefit [22,23]. However, two recent studies have demonstrated that individuals with baseline late-winter 25-hydroxyvitamin D levels <30 nmol/L have ongoing bone loss at a rate of 1%/year when treated with placebo, and that this loss is prevented by vitamin D supplementation [21,24]. When baseline 25-hydroxyvitamin D is >30 nmol/L supplementation is without effect (Figure 2), suggesting 30 nmol/L as a potential definition of deficiency for the purposes of bone health.

### 3.1. Fractures

**Figure 3:**
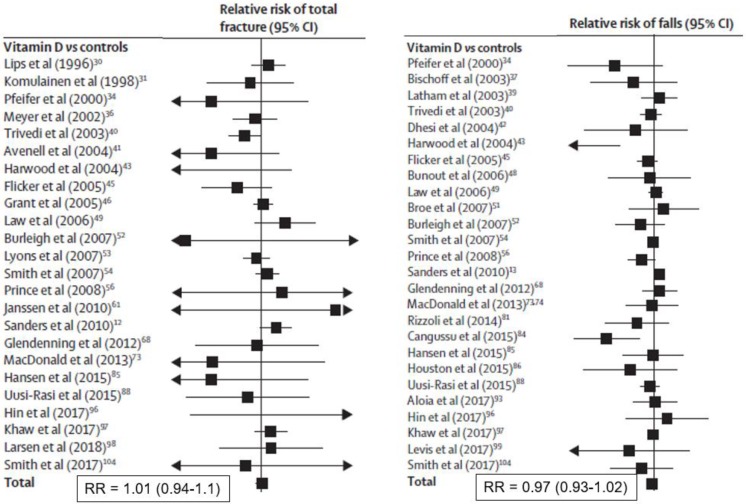
Random effects meta-analyses of effects of vitamin D monotherapy on total fractures (left panel) or falls (right panel) [23]. Eligible studies were randomized controlled trials of adults (>18 years) that compared vitamin D with untreated controls, placebo, or lower-dose vitamin D supplements. Trials with multiple interventions (e.g., co-administered calcium and vitamin D) were eligible if the study groups differed only by use of vitamin D. Studies of hydroxylated vitamin D analogues were excluded. From: Bolland et al., Effects of vitamin D supplementation on musculoskeletal health: a systematic review, meta-analysis, and trial sequential analysis. *Lancet Diabetes Endocrinol* 6: 847-858, 2018 [23]. Used with permission.

Meta-analysis of trials supplementing with vitamin D alone show no effect on total fracture (36 trials, *n* = 44,790, relative risk [RR] 1.00; 95% CI 0.93, 1.07) or hip fracture (20 trials, *n* = 36,655, RR 1.11, 95% CI 0.97, 1.26) [23]. These data are shown in Figure 3, and are consistent with another analysis restricted to community-dwelling adults [5]. The two Chapuy studies of calcium plus vitamin D produced more positive results, as discussed above (Table 1).

### 3.2. Safety

Most studies of vitamin D supplements have used doses of 400–1000 IU/day. These doses have not been associated with evidence of adverse effects, and it is generally held that doses up to 2000 IU/day are safe [25]. However, trials show that vitamin D 4000 IU/day, 60,000 IU/month, or 300,000–500,000 IU/year increase the risk of falls [26,27,28] and/or fractures [26,29], and a recent 3-year study showed that 4000 IU/day and 10,000 IU/day both accelerate bone loss [30]. The threshold for bone benefits of vitamin D (25-hydroxyvitamin D >30 nmol/L) is met with doses of 400–1000 IU/day, so use of these high doses is not justified. Supplement doses greater than 2000 IU/day should only be used in exceptional circumstances, and with appropriate monitoring.

Many observational studies have found that a wide range of diseases (including cardiovascular disease and cancer) are associated with low circulating levels of 25-hydroxyvitamin D. This has led some to hypothesize that vitamin D deficiency contributes to the development of these conditions. The diversity of the conditions showing this association makes it unlikely that vitamin D plays a causative role in their pathogenesis, but large randomized control trials assessing effects of vitamin D supplementation on non-skeletal endpoints have now been conducted. None of the substantive studies has demonstrated beneficial effects [31,32], consistent with earlier meta-analyses of adverse event data from trials with primary bone endpoints [33]. The fact that the populations in these recent studies were not clearly vitamin D-deficient leaves open the question of efficacy of supplements in those with low baseline 25-hydroxyvitamin D levels.

Muscle weakness is a feature of participants with osteomalacia, and low 25-hydroxyvitamin D levels are common in the frail elderly who are frequent fallers. Accordingly, many trials have assessed the effects of vitamin D supplements on muscle function and falls. This literature is contradictory, but the most recent meta-analysis provides no support for a falls benefit from vitamin D (Figure 3) [23]. Vitamin D supplements appear to improve muscle strength, more so in those in institutions and with baseline 25-hydroxyvitamin D levels <30 nmol/L, but not muscle power or mass [34]. However, recent studies in groups selected for vitamin D deficiency have not found benefits to muscle strength [35,36], and one study found that higher-dose supplementation was deleterious to strength and the Timed Up and Go Test [37].

## 4. Do Osteoporosis Drugs Require Co-Administration of Calcium and Vitamin D?

The trials discussed above addressed the value of calcium or vitamin D in people not taking other bone active interventions. It is often pointed out that most studies of pharmaceutical treatments for fracture prevention have co-administered calcium and vitamin D—does this mean that the efficacy of these agents is dependent on that co-administration? It is important to remember that the studies that co-administered calcium and vitamin D did so to both groups, so the differences in bone density or fracture incidence found can be attributed to the drug not to the calcium or vitamin D. It is also important to remember that not all trials of osteoporosis medications have given these supplements. For instance, one large study of alendronate found substantial increases in spine density without co-administration of calcium [38], and another trial formally compared addition of calcium to alendronate and found no benefit to bone density with the addition of calcium [39]. Two large fracture prevention trials using bisphosphonates (clodronate or zoledronate) did not give calcium supplements [40,41] and yet showed fracture prevention comparable to that seen in the many other bisphosphonate trials that did supplement. An early fracture prevention trial with estrogen achieved a positive result without the use of calcium supplements [42], and post hoc analyses of the Women’s Health Initiative showed that hormone treatment reduced fracture numbers whatever the total calcium intake [43]. Thus, results from clinical trials involving 44,000 people indicate that the efficacy of anti-resorptive drugs is not dependent on co-administration of calcium and vitamin D.

However, there are safety considerations that suggest that in some populations, supplements could be justified. Vitamin D deficiency (25-hydroxyvitamin D <25 nmol/L) accelerates bone loss and can result in osteomalacia. Therefore, those with clinical risk factors for vitamin D deficiency (e.g., minimal sunlight exposure, such as the frail elderly and those who are veiled, people with dark skin living at high latitudes) should receive vitamin D replacement—typically with calciferol 400–1000 IU/day, though administration of supplements at weekly or monthly intervals is also safe, effective, and may be more convenient for patients. This is particularly important before the administration of potent anti-resorptive agents, such as intravenous bisphosphonates or denosumab, which can produce marked hypocalcemia in those with severe vitamin D deficiency. Unlike intravenous bisphosphonates, denosumab is safe to use in renal impairment but can cause hypocalcemia in these patients, so calcium supplements are often used to mitigate this risk.

Romosozumab produces rapid increases in bone density resulting in a substantial efflux of calcium from the extracellular fluid to mineralize the new bone being laid down. Calcium supplements were used in the clinical trials of this agent, and its safety and efficacy have not been demonstrated without them.

## 5. Conclusions

The balance of evidence indicates that widespread use of calcium supplements in individuals without a specific bone pathology is not helpful, and may cause harm. Likewise, vitamin D supplements should be reserved for those with clinical risk factors for vitamin D deficiency, principally low sunlight exposure or dark skin and not living in a sunny environment. These recommendations are consistent with those of the United States Preventive Services Task Force, which does not support the use of either calcium or vitamin D supplements in otherwise healthy community-dwelling adults [44]. The position of the International Osteoporosis Foundation is similar, namely that “supplementation with calcium alone for fracture reduction is not supported by the literature” but that “calcium supplementation, with concomitant vitamin D supplementation, is supported for patients at high risk of calcium and vitamin D insufficiency” [45]. The efficacy and safety of anti-resorptive osteoporosis medications does not require co-administration of supplements, other than for the treatment or prevention of vitamin D deficiency in those with clinical risk factors.

## Figures and Tables

**Table 1:** Major trials of calcium ± vitamin D on fracture.

Study	Setting	*n*	Age (years)	Calcium (mg/d)	Vitamin D (IU/d)	Duration (months)	Relative Risk of Fracture	
							Total	Hip
Chapuy 1992, 1994	Nursing home	3270	84 (6)	1200	800	36	0.83 (0.71, 0.97)	0.77 (0.62, 0.96)
RECORD 2005	Community	5292	77 (6)	1000	800	45	0.93 (0.82, 1.06)	1.10 (0.83, 1.47)
Porthouse 2005	Community	3314	77 (5)	1000	800	25	0.96 (0.70, 1.33)	0.71 (0.31, 1.64)
Women’s Health Initiative 2006	Community	36,282	62 (7)	1000	4000	84	0.97 (0.92, 1.03)	0.88 (0.72, 1.07)
Prince 2006	Community	1460	75 (3)	1200	0	60	0.87 (0.69, 1.10)	1.83 (0.68, 4.93)
Reid 2006	Community	1471	74 (4)	1000	0	60	0.92 (0.75, 1.14)	**3.43 (1.27, 9.26)**
Salovaara 2010	Community	3432	67 (2)	1000	800	36	0.83 (0.62, 1.11)	2.00 (0.37, 10.88)

Age data are mean (SD). Relative risks are given with 95% confidence intervals. Where the confidence intervals do not include 1, data are in bold type. Study participants were all female, except in the RECORD study, which was 15% male. Data from Bolland et al., 2015 [4], where the references to the original studies c. Copyright IR Reid, used with permission.
